# Return Home Interviews for Missing Older Adults With Dementia: A Scoping Review

**DOI:** 10.1093/geroni/igab046.551

**Published:** 2021-12-17

**Authors:** Noelannah Neubauer, Elyse Letts, Christine Daum, Antonio Miguel-Cruz, Lauren McLennan, Lili Liu

**Affiliations:** 1 University of Waterloo, University of Waterloo, Ontario, Canada; 2 University of Waterloo, Waterloo, Ontario, Canada; 3 University of Alberta, Edmonton, Alberta, Canada

## Abstract

Introduction: Persons living with dementia are at risk of becoming lost. When a person is returned home safely after a missing incident, an interview with the person or care partner may identify ways to prevent repeat incidents. It is not known if these interviews are being conducted for this population. Objectives: The purpose of this review was to understand return home interviews and whether they are being used with persons who have dementia. Methods: Scholarly and grey literature were searched in 20 databases. Articles were included from any language, year, study design if they included terms resembling “return home interview”, “missing,” “lost,” or “runaway”. Results: Eleven articles in scholarly, and 94 in grey literature sources were included, most from the United Kingdom. The majority of academic (55%) and grey (61%) articles were related to missing children, and none were specifically about persons living with dementia. Interviews were typically conducted within 72 hours after a missing person was returned, and by police or charitable organizations. The main reasons were to understand the causes of the incident and confirm the missing person’s safety, identify support needs, and to provide support to reduce repeat missing incidents. Conclusion: Existing reasons for interviews can also apply to persons with dementia. This review informs future research on return home interviews. It also informs community organizations, and police services interested in adopting this practice with persons living with dementia. Evaluations would confirm if these interviews can reduce repeat incidents and help keep people with dementia safe.

